# The 15th Anniversary of *Materials*—Recent Advances in Advanced Materials Characterization

**DOI:** 10.3390/ma18163767

**Published:** 2025-08-11

**Authors:** Sanichiro Yoshida, Luciano Lamberti, Giuseppe Lacidogna

**Affiliations:** 1 Department of Chemistry and Physics, Southeastern Louisiana University, Hammond, LA 70402, USA; 2 Dipartimento di Meccanica, Matematica e Management, Politecnico di Bari, 70125 Bari, Italy; 3 Department of Structural, Geotechnical and Building Engineering, Politecnico di Torino, 10129 Turin, Italy

Recent developments in materials science and technology have increased the structural complexity of materials and the level of sophistication in describing their properties, especially when the material under analysis is required to exhibit high multi-field performances. Such a scenario entails the need to modify the currently available techniques to characterize material properties accurately, while also considering that each investigated field during multi-field characterization may be conducted at different scales. In general, a well-established method conventionally used for materials at the macroscopic scale may be inapplicable to the same material at the nanoscopic scale. Conventional characterization methods developed for metals may be inappropriate for composite materials or biological specimens. In some cases, either a completely new characterization technique is necessary, or a combination of traditional methods may be sufficient. The characterization protocol must be designed so that the desired information can be gathered reliably and accurately. Analytical/numerical methods may also be critical, though their results should always be corroborated by experimental evidence. The efficient extraction of signals buried in noise may improve the effectiveness of a conventional characterization technique. However, analytical manipulation of signals should not create artifacts that may lead to the misinterpretation of experimental data.

Materials characterization is undoubtedly one of the most studied subjects in science, engineering, and technology. Refs. [1,2,3,4,5,6,7,8,9,10,11,12,13,14,15,16,17,18,19,20] present just a fraction of the examples of the huge amount of research that has been produced on this topic in the last 15 years. Besides manuals, handbooks, and review articles that describe non-destructive materials characterization methods employing different types of electromagnetic, thermal, and acoustic waves/manipulation, specific surveys on the characterization of composite materials, 2D materials, metamaterials, nanomaterials, biomaterials, biotissues, and living cells are also available in the technical literature.

This Special Issue (released on the 15th anniversary of MDPI’s journal *Materials*) focuses on the recent advances in the characterization of advanced materials. The Special Issue includes 1 review article and 15 research articles covering various aspects of materials applications, such as the following: (i) data extraction and processing; (ii) design and characterization of new materials with optimized properties; (iii) investigations on mechanical, electromagnetic, and optical properties of new materials; (iv) evaluation of superalloy parts produced by additive manufacturing; (v) materials for biomedical uses; (vi) environmental applications; (vii) energy production; and (viii) analysis and preservation of artistic treasures.

The first issue of paramount importance in materials characterization is determining how to obtain detailed 3D information on a material’s structure. The 3D reconstruction of microscopic structures with a nanometric resolution is a very challenging task. In this regard, Mura et al. [21] reviewed the FIB-SEM tomography technique combining Focused Ion Beam (FIB) [22,23] and Scanning Electron Microscopy (SEM) [24,25]. FIB-SEM tomography bridges the non-destructive X-ray families of tomographic techniques (providing a submicron resolution) with the nano- to atomic-scale resolution achieved by Transmission Electron Microscopy (TEM) tomography [26,27]. This is proven by the extensive survey conducted in Ref. [21] on the most relevant applications of FIB-SEM for fuel cells, batteries, solar cells, nuclear energy, metal alloys, ceramics, fibrous materials, and earth sciences.

Data processing is also fundamental in materials characterization, especially when data from different sources covering a broad spectrum of spatial and temporal scales may interfere by increasing noise. Deconvolution can solve this issue, for example, through application in the analysis of strain maps derived from materials subjected to mechanical testing: deconvolution reduces noise, making it possible to recover actual displacement and strain fields from localized digital image correlation maps or localized spectrum analyses [28]. In Ref. [29], Speranza illustrated the application of deconvolution in analyzing Auger spectra obtained through the X-ray Photoelectron Spectroscopy (XPS) of carbon. The principles of Auger spectra/spectroscopy and the XPS technique are, respectively, detailed in Refs. [30,31] and Refs. [32,33].

The optimization of material properties (also including the optimization of processing parameters to improve material properties) and investigation of properties for newly developed materials represent the backbone of materials characterization. In this regard, Konieczny et al. [34] optimized the mechanical (i.e., Vickers hardness) and electrical properties (i.e., conductivity) of CuNi_2_Si_1_ by combining experimental approaches and metaheuristic algorithms. Hardness and conductivity were fitted as quartic polynomial functions with respect to aging temperature and aging duration via the factorial plan of experiments approach. Classical metaheuristic optimization methods such as genetic algorithms (GAs) [35], gray wolf optimization (GWO) [36], particle swarm optimization (PSO) [37], student psychology-based optimization (SPBO) [38], teaching–learning-based optimization (TLBO) [39], and the whale optimization algorithm (WOA) [40] were then used to minimize the difference between the desired properties and the fitted properties. The above-described approach was successfully utilized for both undeformed and cold-rolled CuNi_2_Si_1_ specimens, thereby providing realistic values of aging parameters.

Fotouhiardakani et al. [41] employed XPS, Fourier-transform infrared spectroscopy (FTIR) [42,43], and profilometry [44] to analyze the chemistry and growth rate of a coating deposited on a fluoropolymer. Deposition was conducted using dielectric barrier discharge at atmospheric pressure, employing an oxygen-containing organic precursor in a nitrogen environment. The fragmentation process and the growth mechanisms of the coating were optimized with respect to the total flow, precursor concentration, and precursor residence time. Rosic et al. [45] combined differential thermal analyses (DTAs) [46], X-ray diffraction (XRD) [47,48], FTIR, energy-dispersive X-ray spectroscopy (EDX) [49,50], field emission Scanning Electron Microscopy (FESEM) [25,51], and the nitrogen adsorption method [52,53] to analyze the composition and morphology of a molybdenum-based ceramic nanostructured material, such as Co_0.9_R_0.1_MoO_4_; an important novel aspect of Ref. [45] was the use of the glycine nitrate process to synthesize Co_0.9_R_0.1_MoO_4_ nanoparticles.

De Giorgi [54] designed a kirigami-based metamaterial with tailored optical properties that improved common camouflage techniques so as to yield a product that was cheap, light, and easy to manufacture and assemble. A typical kirigami structure geometry is based on rotating squares [55]. Light polarization and birefringence [56] were successfully exploited in [54] to obtain transparency and color-changing properties using two polarizers and common cellophane tape. The electromagnetic properties of advanced materials also were investigated in this Special Issue. In particular, Camacho Hernandez and Link [57] presented an innovative method for estimating the effective permittivity of anisotropic fibrous media and disclosing the orientation and microstructure of fibers. They integrated the method formulated in their previous work with modeling theories of structural anisotropy and wave propagation in anisotropic media [58,59,60]. Interestingly, the resonance frequency of a woven alumina fabric in a microwave resonator, determined experimentally, was consistent with its counterpart evaluated numerically by setting the fabric’s permittivity to the values provided by the proposed approach. In Ref. [61], Dapor studied the differences in the elastic scattering spectra of electrons and positrons in amorphous low-density polyethylene, focusing on the underlying mechanisms that influence spectral features. Elastic Peak Electron Spectroscopy (EPES) [62,63] was used to isolate key factors such as recoil energy, Doppler broadening, and the interplay between elastic and inelastic mean free paths. Monte Carlo simulations [64] were used to systematically compare the elastic scattering interactions of electrons and positrons with polyethylene.

Johnson and Kujawski [65] characterized the notch sensitivity of additively manufactured Inconel 718 parts produced by laser powder bed fusion. Three different root radii were evaluated under tensile conditions for V-notched test specimens and smooth specimens built in vertical and horizontal orientations. Both the total axial strain and localized notch diametral strain were measured. Finite element simulations were in agreement with the actual strain measurements near the notch.

Some important fields that are well documented in this Special Issue are the synthesis and characterization of new materials for biomedical use, environmental applications, and energy production (including fuel cells and renewable energy sources). For example, Wu et al. [66] successfully prepared hydroxyapatite (calcium phosphate, HA) using a precipitation method with eggshell as a raw material. HA is an important material in biomedical applications because it closely resembles human bones. The HA powder synthesized in Ref. [66] was press-formed and sintered at various temperatures (in the range of 800–1400 °C, which has never been conducted before) to investigate the impact of sintering temperature on the mechanical properties, such as hardness, compressive strength, and fracture toughness, of the sintered HA samples (E-HA). The phase content and crystallinity of the sintered E-HA samples were analyzed with XRD [47,48] while the sample microstructure was observed with FESEM [25,51]. The bacterial culture experiments conducted on sintered E-HA indicated that it possessed significant antibacterial efficacy against the Streptococcus mutans, thus highlighting the potential of eggshell-derived HA as an effective material for biomedical applications.

Cho et al. [67] demonstrated the potential application of green-chemically synthesized silver nanoparticles (AgNPs) as selective antibacterial agents. For that purpose, stable AgNPs were biologically synthesized using common walkingstick (*Diapheromera femorata*) aqueous extract. AgNPs were then UV-treated and tested as antibacterial agents to inhibit the growth of four pathogenic bacteria (*Burkholderia cenocepacia* K-56, *Klebsiella pneumoniae* ST258, *Pseudomonas aeruginosa* PAO1, and *Staphylococcus aureus* USA300), as well as one common bacterium (*Escherichia coli* BW25113). Remarkably, UV-treated AgNPs significantly and selectively inhibited the growth of *Staphylococcus aureus* USA300 and *P. aeruginosa* PA01. The optimal duration of UV exposure yielding the strongest antibacterial activity was also investigated in Ref. [67].

Regarding environmental applications, Kurbonov et al. [68] studied new materials for water remediation. They prepared mesoporous silica sieves through sol–gel synthesis using diester gemini surfactants as pore templates. Submicron-size mesoporous spherical silica particles were prepared in an alkali-catalyzed reaction using a tetraethyl orthosilicate precursor and bis-quaternary ammonium gemini surfactants with diester spacers of varied lengths as pore-forming agents. The effect of the spacer length on the particle morphology was studied using nitrogen porosimetry [52,53], small-angle X-ray scattering (SAXS) [69,70], ultra-small-angle neutron scattering [71,72], and Scanning and Transmission Electron Microscopy (SEM, TEM) [24,27]. The new materials were tested for the adsorption of Pb(II) in a batch sorption experiment and demonstrated a higher adsorption capacity than that of most silica-based sorbents reported in the recent literature.

An example of the application of advanced characterization methods to renewable energy sources documented in this Special Issue is the study by Lejda et al. [73] that used TGA/DTA-QMS (thermogravimetry [74,75] coupled with thermal analysis [46] and quadrupole mass spectroscopy [76,77]) to assess the oxidation susceptibility of a pool of nanocrystalline powders of the semiconductor kesterite Cu_2_ZnSnS_4_ for prospective photovoltaic applications. The Cu_2_ZnSnS_4_ powders were prepared via a mechanochemically assisted synthesis route from two precursor systems.

Polymer electrolyte membrane fuel cells (PEMFCs) are recognized as the most suitable energy conversion device for next-generation zero-emission electric vehicles. In this context, Yoo et al. [78] analyzed the complex relationships between catalyst degradation and binder performance in high-power PEMFCs with the goal of developing more durable PEMFC components. The study by Ref. [78] allowed for the existing limitations on assessing binder durability to be overcome, and its degradation in situ during the accelerated stress test process was measured. Scanning Electron Microscopy/energy-dispersive spectroscopy (SEM-EDS) [25,79] analysis measured the degradation rates for the catalyst, the support, and the binder. The method assessing the distribution of relaxation times served to measure the increase in oxygen reduction reaction resistance and decrease in proton transport resistance in situ.

The last two papers of this Special Issue focused on the analysis and preservation of artistic treasures. Wu et al. [80] combined energy-dispersive X-ray fluorescence (EDXRF) [81,82], ultra-depth-of-field optical microscopy [83,84], SEM-EDS [25,79], Raman spectroscopy [85,86], and XRD [47,48] to characterize the body and glaze chemical composition, microstructure, and crystalline phases present in high-temperature iron-series glazed wares produced in the Guangyuan kiln during the Song Dynasty (China, 960–1279 A.D.). The study elucidated the compositional characteristics, structural features, and color formation mechanisms of these wares, thereby revealing the compositional and structural variations in ancient Chinese high-temperature iron-series glazed wares and the chemical state of the iron within the glaze matrix.

Colomban et al. [87] analyzed Mīnā’ī decorations. These wares, produced in Persia during the 12th and 13th centuries, are regarded as the first sophisticated painted enamel decorations created by potters, and are considered to be among the most luxurious in the Islamic world. Due to the thinness of these enamel layers, their detailed characterization remains challenging, even with the use of advanced techniques, such as Proton-Induced X-ray Emission (PIXE) [88] analysis and Rutherford Backscattering Spectrometry (RBS) [89]. To solve this issue, Ref. [87] presented the first combined noninvasive analysis ever performed on Mīnā’ī wares by using XRF [81,90] and Raman spectroscopy [85,86]. İznik shards (from the 17th century), which feature similarly styled but thicker enamel decorations, were also analyzed for comparison. Interestingly, the Mīnā’ī paste was found to contain lead and tin, suggesting the use of a lead-rich frit in its composition. This finding was confirmed by SEM–EDS micro-destructive analysis.

The variety of topics covered by this Special Issue demonstrates the broadness of the applications of advanced materials characterization methods, fitting very well into all fields of science, arts, engineering, and technology. The need to combine several experimental protocols to optimize the analysis of materials is fully confirmed by most of the papers included in this Special Issue. Characterization methods for advanced materials (including those used for creating artistic treasures) present high levels of standardization that can be tailored to each specific investigation scale. This ability is very useful in optimizing all stages of product life, from materials characterization to design verification, including the monitoring/improvement of production processes and the evaluation of product quality. Also, it helps to preserve artistic treasures by providing detailed information on composition, state, crafting, and the materials utilized in ancient times.

It should be noted that the studies included in this Special Issue made marginal use of artificial intelligence (AI) techniques in the materials characterization process. AI is expected to become a standard in materials characterization within a few years (see, for example, Refs. [19,91,92,93]). In fact, AI may greatly help analysts to interpret and extract meaningful insights from large and complex datasets generated by experimental techniques such as, for example, SEM, TEM, XRD, Raman spectroscopy, and XPS spectra for surface composition. Furthermore, AI can be used to predict material properties by creating digital twins of experimental campaigns. AI-based materials characterization approaches should certainly be the subject of a future Special Issue of *Materials*.
